# Recent progress on the genetics and molecular breeding of brown planthopper resistance in rice

**DOI:** 10.1186/s12284-016-0099-0

**Published:** 2016-06-14

**Authors:** Jie Hu, Cong Xiao, Yuqing He

**Affiliations:** Key Laboratory of Ministry of Education for Genetics, Breeding and Multiple Utilization of Crops, Fujian Agriculture and Forestry University, Fuzhou, 350002 China; National Key Laboratory of Crop Genetic Improvement and National Center of Crop Molecular Breeding, Huazhong Agricultural University, Wuhan, 430070 China

**Keywords:** Gene pyramiding, Marker assisted backcross breeding, *Nilaparvata lugens*, Resistance genes, *Oryza sativa*

## Abstract

Brown planthopper (BPH) is the most devastating pest of rice. Host-plant resistance is the most desirable and economic strategy in the management of BPH. To date, 29 major BPH resistance genes have been identified from *indica* cultivars and wild rice species, and more than ten genes have been fine mapped to chromosome regions of less than 200 kb. Four genes (*Bph14*, *Bph26*, *Bph17* and *bph29*) have been cloned. The increasing number of fine-mapped and cloned genes provide a solid foundation for development of functional markers for use in breeding. Several BPH resistant introgression lines (ILs), near-isogenic lines (NILs) and pyramided lines (PLs) carrying single or multiple resistance genes were developed by marker assisted backcross breeding (MABC). Here we review recent progress on the genetics and molecular breeding of BPH resistance in rice. Prospect for developing cultivars with durable, broad-spectrum BPH resistance are discussed.

## Introduction

Rice is the most important cereal crops in the Asia-Pacific region, particularly China, India, Japan, Indonesia, and Vietnam, where the brown planthopper (BPH, *Nilaparvata lugens* Stål) has become its most damaging insect pest. In 2005 and 2008 China reported a combined rice production loss of 2.7 million tons due to direct damage caused by BPH (Brar et al. 2009). Currently, the main method of controlling BPH is application of pesticides such as imidacloprid. However, the intensive and indiscriminate use of chemicals leads to environmental pollution, kills natural enemies of the target pest, may result in development of BPH populations that are resistant/tolerant to insecticides, ultimately leading to a resurgence in BPH populations (Lakshmi et al. 2010; Tanaka et al. 2000). Host-plant resistance is therefore most desirable and economic strategy for the control or management of BPH (Jena et al. 2006).

BPH is a migratory, monophagous rice herbivore. According to the length of the wing, adults BPH are biomorphic with varying wing lengths. The short winged cannot migrate, but produces larger amounts of eggs; BPH with long wings are able to fly between regions and bridge gaps in subsequent cropping seasons. The combined effect of the two types makes BPH an internationally explosive and devastating pest of rice. The differentiation of wing type is genetically controlled and a research group at Zhejiang University recently identified two highly homologous insulin receptor genes that play a key role in the wing differentiation (Xu et al. 2015).

Different biotypes (or races) of BPH vary in virulence (or ability to infest) different rice genotypes (Sogawa 1978). Four biotypes have been well known since the 1980s. In China, biotype 2 dominates, from the 1990s has sometimes been mixed with biotype 1 (Tao et al. 1992). However, the current population may be shifting to the more destructive Bangladesh type (Lv et al. 2009). New biotypes arise to overcome resistance genes prolonged use in a single widely used variety or suite of varieties with the same resistance gene (Cohen et al. 1997; Jing et al. 2012). For example, the first resistant variety IR26 possessing the *Bph1* gene became susceptible of biotype 2 after only two years of use (Khush 1971). The genetic mechanism of BPH biotype generation in BPH is still not well understood, but there is overwhelming evidence from many plant disease/pest combinations that virulence involves the change or loss of specific effector proteins that are recognized by the plant host to induce the resistance (antibiosis) response.

Rice varieties have different mechanisms of resistance to BPH, classed as antixenosis, antibiosis and tolerance (Alam and Cohen 1998; Painter 1951). Antibiosis is the most commonly studied mechanism (Cohen et al. 1997; Du et al. 2009; Qiu et al. 2010). BPH behavior (host-searching, feeding, mating) is most obviously affected by resistant varieties through antibiosis. After infestation by BPH the rice plant activates its own stress response for defense, including secretion of insect-toxic compounds, activation of expression of genes producing metabolic inhibitors, and formation of physical barrier (such as cuticle thickening, and callose deposition) to prevent continuous feeding by BPH (Cheng et al. 2013). Hao et al. (2008) showed that plants carrying *Bph14* undergo quicker deposition of callose on the sieve plate following infestation than those without the gene, suggesting that sieve tube plugging is an important mechanism for defense to BPH.

Since the development of molecular markers (SSR, InDel, SNPs) and functional genomics, the genetic studies of BPH resistance in rice have intensified. To date 29 BPH resistance genes have been detected in rice, and four (*Bph14*, *Bph26*, *Bph17* and *bph29*) have been cloned (Du et al. 2009; Liu et al. 2015; Tamura et al. 2014; Wang et al. 2015). Both marker-assisted selection (MAS) and conventional breeding have enabled resistance genes to be combined (or ‘pyramided’) in elite rice varieties to improve BPH resistance and its durability. We review here recent progress on BPH resistance genetics and molecular breeding in rice, aiming to help a wider utilization of BPH resistance genes.

## Review

### BPH resistant germplasms

#### Evaluation of BPH resistance

A thorough evaluation of BPH resistance in the abundant germplasms is critical for identification and utilization of BPH resistance genes (Jena and Kim 2010). Various evaluation methods were developed to measure response to BPH in rice varieties. Based on the type host:pest interaction, evaluation methods can be divided into two groups. The first directly evaluates host resistance by measuring the degree of damage following BPH infestation. The modified seedbox test (SSST test) is recognized as a standard method. SSST assesses damage to seedlings (leaf yellowing, plant withering and dwarfing) caused by the progeny of an initial infestation with a set number of nymphs (Panda and Khush 1995). It is suitable time- and space-saving assay for testing of germplasm and breeding materials. However, results from this test are affected by temperature, humidity, nymphs instar, density, biotype, population and natural enemies. The second approach indirectly the relative host response by examining the physiological and biochemical reactions of the BPH (feeding rate, fecundity and survival) feeding on different varieties. Parameters measured include honeydew excretion, survival rates, preference settling, and feeding behavior (Pathak et al. 1982; Sangha et al. 2008; Klignler et al. 2005). Some t evaluation methods attempt to address host tolerance using compensation ability and yield loss rate (Dixon et al. 1990; Alam and Cohen 1998). Ultimately, all possibilities for reducing the insect population or its fitness through use host genotype must be reconfirmed in laboratory/greenhouse trials and in the field.

#### Source of BPH resistance

**Fig. 1:**
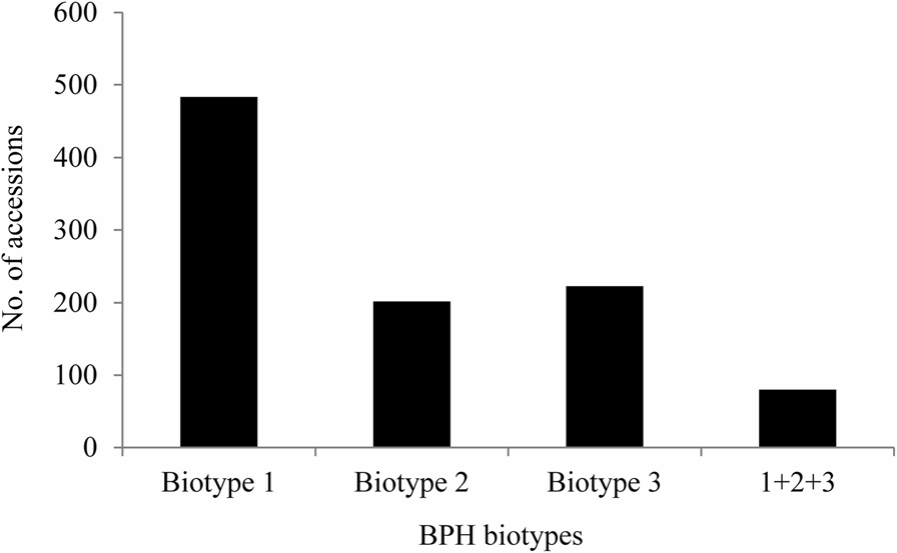
Frequencies of rice accessions resistant to different BPH biotypes. The data were partially selected and summarized from the Genesy database (https://www.genesys-pgr.org/zh/welcome). The total of 573 cultivated rice accessions showed resistance to at least one biotype. *Biotype 1*, *Biotype 2* and *Biotype 3* represent the number of cultivars only resistance to biotype1, 2 or 3 of BPH, respectively. *1 + 2 + 3* denotes the number of cultivars resistance to all three biotypes

**Fig. 2:**
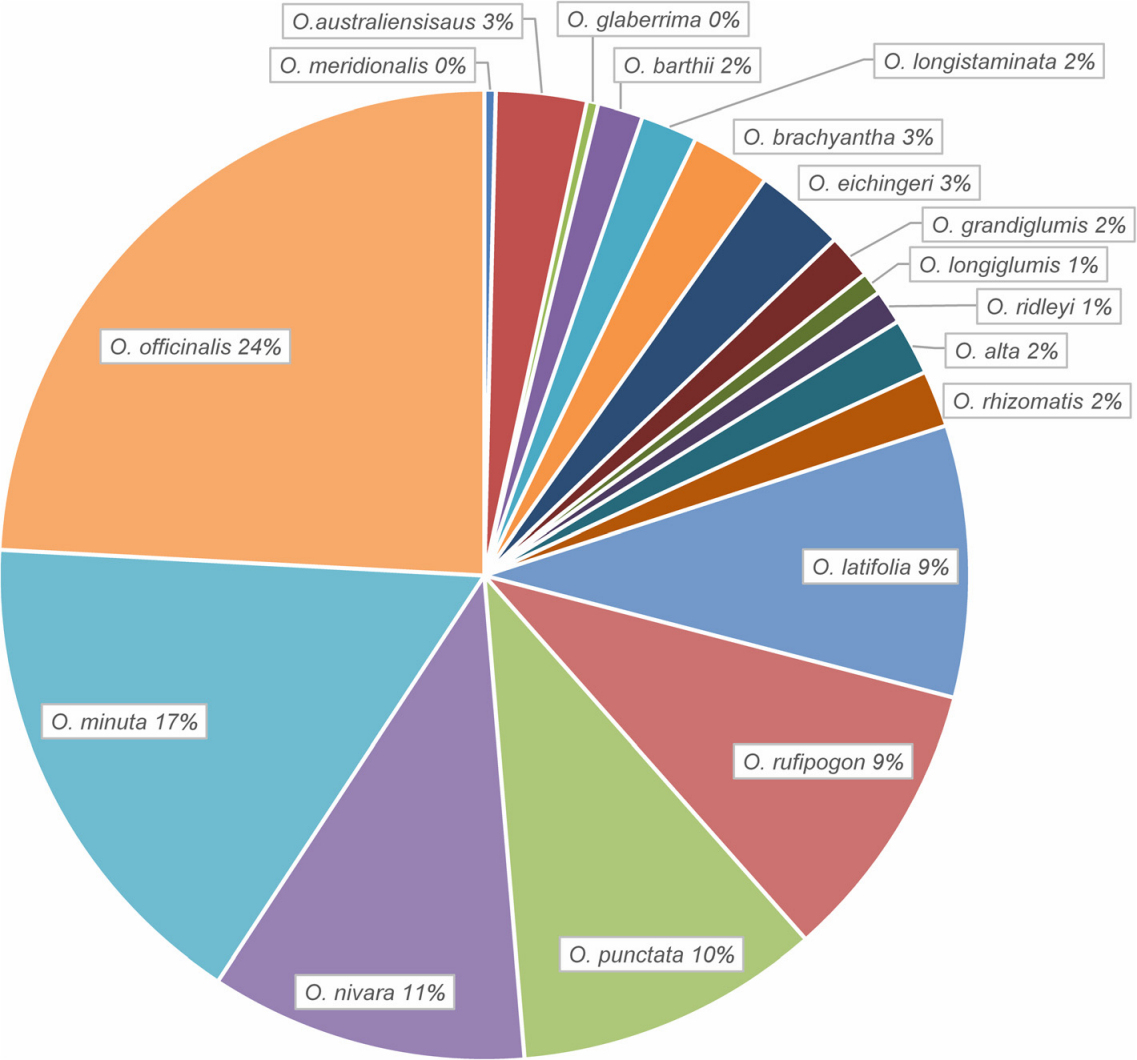
Frequencies of wild rice species accessions with resistance to BPH at IRRI. Data are summarized from a search of the Genesy database; 265 accessions (involving 18 species) showed high resistance to all three BPH biotypes

Since the 1970s, a large number of germplasm accessions have been screened for response to BPH at the International Rice Research Institute (IRRI) by mass screening evaluation (Jackson 1997). After searching the Genesy database (https://www.genesys-pgr.org/zh/welcome) maintained at IRRI we identified a total of 573 cultivated rice accessions that showed resistance to at least one BPH biotype. Among them, 484 accessions (92.5 %) showed resistance to biotype 1, and only 80 accessions (15.3 %) were resistant to all three biotypes (Fig. 1). Wild rice is a key source of resistant germplasm. Various species commonly show high resistance to all three biotypes. Eighteen species of wild rice, comprising 265 accessions, were highly resistant, and two species (*O. officinalis* and *O. minuta*) accounted for 41 % of the total (Fig. 2).

The first BPH resistance was identified in 1967 (Pathak et al. 1969). Since then genes *Bph1*, *bph2*, *Bph3* and *bph4* have been identified in genetic analyses of various donors (Lakashminarayana and Khush 1977; Khush et al. 1985). These four genes have been used extensively in breeding programs in Southeast Asia (Jairin et al. 2007a), and a large number of BPH resistant varieties have been released by IRRI since 1976. However, some of them have lost effectiveness with the evolution and subsequent increase of new biotypes (Table 1).

**Table 1:** Resistance of Asian cultivars carrying BPH resistance genes

Name	ACC^a^	Origin^b^	Gene	RS^c^	RL^d^
MGL 2	6218	IND	*Bph1*	3.00	R
MTU15	6365	IND	*Bph1*	9.00	S
IR 28	30411	PHL	*Bph1*	4.00	MR
IR 29	30412	PHL	*Bph1*	7.47	MS
IR 30	30413	PHL	*Bph1*	7.14	MS
IR 34	30415	PHL	*Bph1*	4.12	MR
IR 26	24154	PHL	*Bph1*	2.83	R
IR 44	39341	PHL	*Bph1*	2.56	R
IR 46	32695	PHL	*Bph1*	8.57	S
ASD9	6380	IND	*bph2*	9.00	S
PTB18	11052	IND	*bph2*	1.80	HR
IR 32	30414	PHL	*bph2*	5.00	MS
IR 38	32536	PHL	*bph2*	2.65	R
IR 40	36958	PHL	*bph2*	5.59	MS
IR 42	36959	PHL	*bph2*	3.37	MR
IR 36	39292	PHL	*bph2*	2.50	R
IR 54	55969	PHL	*bph2*	5.92	MS
GANGALA	15259	LKA	*Bph3*	3.01	MR
MUDUKIRIEL	15719	LKA	*Bph3*	5.46	MS
HONDERAWALA	31415	LKA	*Bph3*	1.86	HR
KURU HONDARAWALU	36303	LKA	*Bph3*	3.00	R
MUTHUMANIKAM	40850	LKA	*Bph3*	1.46	HR
BABAWEE	8978	LKA	*bph4*	1.50	HR
VELLAI ILLANKALI	15233	LKA	*bph4*	4.12	MR
HEENHORANAMAWEE	15286	LKA	*bph4*	3.24	MR
KAHATA SAMBA	15297	LKA	*bph4*	3.57	MR
GAMBADA SAMBA	15406	LKA	*bph4*	3.69	MR
LEKAM SAMBA	15412	LKA	*bph4*	2.92	R
SULAI	15421	LKA	*bph4*	3.25	MR

The rice varieties or lines was selected from Genesy database (https://www.genesys-pgr.org/zh/welcome) in IRRI. The information of genes that these lines carry was described as Ali and Chowdhury (2014) and Jena and Kim, (2010). The resistance data of these lines were obtained in our previous study of seeding resistance

^a^Accession numbers in the IRRI genebank

^b^IND (India), PHL (Philippines), LKA (Sri Lanka)

^c^Resistance scores at seedling stage

^d^Resistance level, HR (highly resistant), R (resistant), MR (moderately resistant), MS (Moderately susceptible), S (susceptible)

### Genetics of BPH resistance

#### Mapped BPH resistance genes

Twenty nine BPH resistance genes have been identified from ssp. *indica* and wild relatives (Ali and Chowdhury 2014; Wang et al. 2015). Most of these genes were located to specific rice chromosome regions, but the identities of a few (e.g. *bph5* and *bph8*) are confusing because of the lack marker technology in early studies (Qiu et al. 2014). Since the development of molecular markers (such as SSR, InDel, and SNPs) and functional genomics increasing numbers of resistance genes have been fine mapped and some were cloned. To date, more than ten genes have been fine mapped to regions of less than 200 kb (Table 2). Most of resistance alleles are dominant, but several are a few are recessive (*bph4*, *bph5*, *bph7*, *bph8*, *bph19* and *bph29*).

**Table 2:** Chromosome locations of BPH resistance genes/QTLs in rice

Gene/QTL	chr	Position (Mbp)	Donor	References
*Bph1*	12	13.10–13.28	Mudgo, TKM6	Kim and Sohn 2005
	12 L	22.81–22.93	Mudgo	Cha et al. 2008
	12 L	24.00–25.00	Nori-PL3	Sharma et al. 2002
*bph2*	12 L	22.13–23.18	IR1154-243	Murai et al. 2001
	12 L	13.21–22.13	ASD7	Sun et al. 2006
*Bph26/bph2*	12 L	22.87–22.88	ADR52	Tamura et al. 2014
*bph7*	12 L	19.95–20.87	T12	Qiu et al. 2014
*Bph9*	12 L	19.11–22.13	Kaharamana	Su et al. 2006
	12 L	19.00–22.50	Pokkali	Murata et al. 2001
*Bph10(t)*	12 L	19.00–23.00	IR65482-4-136, *O. australiensis*	Ishii et al. 1994
*Bph18(t)*	12 L	22.25–23.48	IR65482-7-216, *O. australiensis*	Jena et al. 2006
*Bph21(t)*	12 L	23.28–24.41	IR71033-121-15, *O. minuta*	Rahman et al. 2009
*Bph12*	4S	5.21–5.66	B14, *O. latifolia*	Qiu et al. 2012
*Bph15*	4S	6.68–6.90	B5, *O. officinalis*	Lv et al. 2014
*QBph4.1*	4S	6.70–6.90	IR02W101, *O. officinalis*	Hu et al. 2015a
*QBph4.2*	4S	6.58–6.89	IR65482-17-511, *O. australiensis*	Hu et al. 2015b
*Bph17*	4S	6.93–6.97	Rathu Heenati	Sun et al. 2005
*Bph20(t)*	4S	8.20–9.60	IR71033-121-15, *O. minuta*	Rahman et al. 2009
*Bph6*	4 L	21.36–21.39	Swarnalata	Qiu et al. 2010
*Bph27*	4 L	19.12–19.20	GX2183, *O. rufipogon*	Huang et al. 2013
*Bph27(t)*	4 L	20.79–21.33	Balamawee	He et al. 2013
*bph12(t)*	4 L	20.20–21.20	*O. officinalis*	Hirabayashi et al. 1999
*bph11(t)*	3 L	35.60–35.80	*O. officinalis*	Hirabayashi et al. 1998
*Bph14*	3 L	35.70–35.72	B5, *O. officinalis*	Du et al. 2009
*QBph3*	3 L	35.63–35.67	IR02W101, *O. officinalis*	Hu et al. 2015a
*Bph13*	3S	5.18–5.70	IR54745-2-21, *O. officinalis*	Renganayaki et al. 2002
*bph19*	3S	7.18–7.24	AS20-1	Chen et al. 2006
*qBph3*	3	18.27–20.25	Rathu Heenati	Kumari et al. 2010
*Bph3*	6S	1.21–1.40	Rathu Heenati	Jairin et al. 2007b
*bph4*	6S	1.20–1.76	Babawee	Kawaguchi et al. 2001
*Bph25*	6S	0.20–1.71	ADR52	Myint et al. 2012
*bph29*	6S	0.48–0.49	RBPH54, *O. rufipogon*	Wang et al. 2015
*Bph6*	11	17.23–18.27	IR54741-3-21-22, *O. officinalis*	Jena et al. 2003
*Bph28(t)*	11	16.90–16.96	DV85	Wu et al. 2014

All BPH resistance genes identified to date are from *indica* varieties and wild relatives. *Bph1*-*Bph9*, *Bph19*, *Bph25*-*Bph28* are from *indica* accessions, wheraes *Bph10*-*Bph18*, *Bph20*, *Bph21*, *Bph27* and *bph29* are from wild rice species (Table 2). Introgression lines (ILs) derived from crosses of *O. sativa* and wild species have been used to map many of the BPH resistance genes (Jena and Khush 1990; Brar and Khush 1997). For example, *Bph18*, located on 12 L, was identified in IR65482-17-216-1-2, a BPH resistant IL derived from *O. australiensis*. Up to now, 11 genes have been identified in wild rice, including *Bph11*-*Bph15* were from *O. officinalis*, *Bph10* and *Bph18* were from *O. australiensis*, *Bph20* and *Bph21* were from *O. minuta*, and *Bph27* and *bph29* were from *O. rufipogon*.

**Fig. 3:**
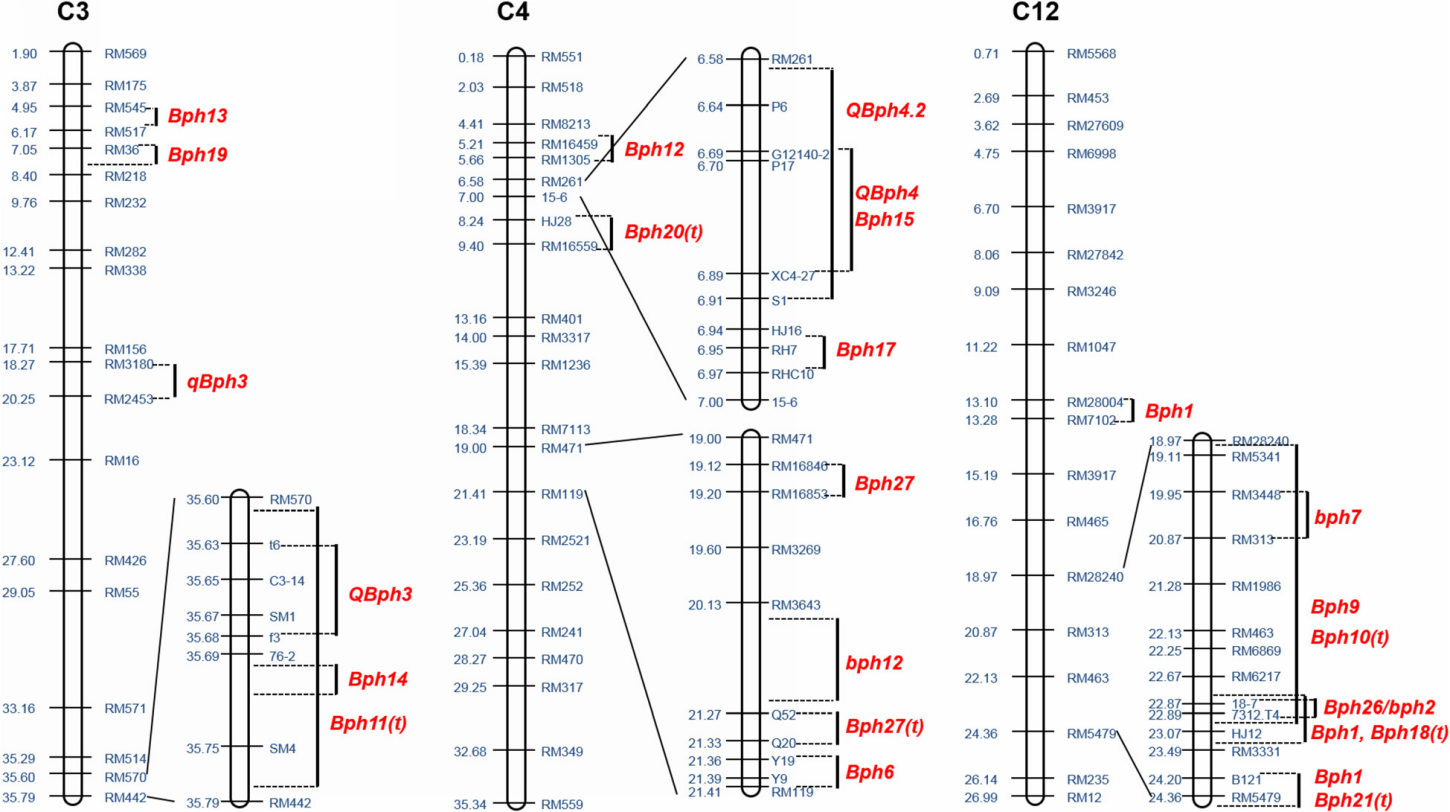
Locations of BPH resistance genes on rice chromosomes

Multiple BPH resistance genes are clustered in a similar way to blast resistance genes (Jena and Kim 2010; Ramalingam et al. 2003). For example, eight genes (*Bph1*, *Bph2*, *Bph9*, *Bph10*, *Bph18*, *Bph19*, *Bph21* and *Bph26*) are cluster in a 22–24 Mb region on chromosome 12 L, and six (*Bph12*, *QBph4*, *QBph4.2*, *Bph15*, *Bph17* and *Bph20*) are closely linked in a region of 5–9 Mb on chromosome 4S. Another three genes together are located within a19–22 Mb on chromosome 4 L, and four are concentrated in a 0–2 Mb region on chromosome 6S (Table 2, Fig. 3). These QTLs/gene clusters might involve different genes, different alleles at a single locus, or even the same gene, but mediate different resistance mechanisms or show different response to different BPH biotypes (Qiu et al. 2010). Additional genetic analyses, including allelism tests and gene cloning are needed to resolve these possibilities.

Multiple BPH resistance genes/QTLs with the same names are also located to different positions. For example, *Bph1* from three different donors (Mudgo, TKM6 and Nori-PL3) was mapped to different positions on chromosome 12 (Table 2). *Bph26* was recently cloned and sequence comparison indicated that it is the same as *Bph2* (Tamura et al. 2014). Discrepancies in genetic maps have caused duplicated nomenclature for the same gene. For example, *Bph27* and *Bph27(t)* were fine mapped to the adjacent locations on the long arm of chromosome 4 (Huang et al. 2013; He et al. 2013), and it is possible that they might be different due to their different origins (derived from wild rice and a cultivated relative, respectively). According to the rules of genetic nomenclature for rice, it is necessary for the authors of different reports to rename duplicated genes to avoid confusion to readers. *Bph3* and *Bph17* each described as single Mendelian factors in the resistant cultivar Rathu Heenati (RH) by different research groups. The rice scientific community has accepted the findings as *Bph17* on chromosome 4 (Rahman et al. 2009, Qiu et al. 2012) and *Bph3* on chromosome 6 (Jairin et al. 2010, Myint et al. 2012). These reports acknowledged in review papers (Jena and Kim, 2010, Fujita et al. 2013, Cheng et al. 2013) and on the cereal crop GRAMENE website (http://archive.gramene.org/documentation/nomenclature/) as well as Oryzabase (http://www.shigen.nig.ac.jp/rice/oryzabase/). However, Liu et al. (2015) reported the gene chromosome 4 cloned from RH as’*Bph3*’ when it actually originally reported as ‘*Bph17*’ (Sun et al. 2005). In our opinion the cloned gene on chromosome 4 (Liu et al., 2015) should have been reported as ‘*Bph17*’.

#### Mapping of minor BPH resistance QTLs

Using different mapping populations (RIL, DH, F_2:3_) from crosses of susceptible and resistant varieties, more QTLs were detected on all rice chromosomes except 5 and 9 (Alam and Cohen, 1998; Soundararajan et al. 2004; Liu et al. 2009; Ali and Chowdhury, 2014). However, those minor QTLs could not be confirmed due to the complex inheritance of the BPH resistance (Jena and Kim, 2010). Several studies showed that some highly resistant varieties carry many minor QTLs in addition to one or more major genes. Such combinations suggest possibilities for more durable resistance contributed by minor QTLs (Bosque-Perez and Buddenhagen 1992). For example, an elite variety IR64 from IRRI showed more durable and stable resistance than IR26, although both carry *Bph1*. In a further seven minor QTLs were detected on chromosomes 1, 2, 3, 4, 6 and 8 from IR64 (Alam and Cohen 1998). Likewise, the Sri Lankan variety Rathu Henati has shown durable resistance to all four BPH biotypes in Southeast Asia since the 1970s, as it not only carries major genes *Bph3* and *Bph17*, but also minor QTLs on chromosomes 2, 3, 4, 6 and 10 (Jairin et al. 2007a; Kumari et al. 2010; Sun et al. 2005). A recent study showed that *indica* cultivar ADR52 possesses two major genes *Bph25* and *Bph26*, along with several minor QTLs associated with resistance to BPH, white-backed planthopper (WBPH) and green leafhopper (Srinivasan et al. 2015).

#### Map based cloning of BPH resistance genes

Gene identity helps to clarify the molecular mechanisms of BPH resistance. Advances in sequencing technology and functional genomics have facilitated BPH resistance gene cloning. To date, *Bph14*, *Bph26*, *Bph17* and *bph29* have been cloned by map-based cloning.

*Bph14* is the first cloned BPH resistance gene originated from *O. officinalis. Bph14* was originally fine-mapped to a 34 kb region on chromosome 3 L. Sequence comparison base on two parents showed that gene *Ra* was unique to the resistant parent. Further genetic complementation tests determined that *Ra* was the *Bph14*, which encodes a coiled-coil, nucleotide-binding and leucine-rich repeat (CC-NB-LRR) protein. The unique LRR domain might function in specific recognition of a BPH effector, with consequent activation of the defense response, possibly through induction of a SA-dependent resistance pathway (Du et al. 2009).

*Bph26* was cloned from *indica* variety, ADR52. Early study showed that ADR 52 carries two genes, *Bph25* and *Bph26* located on chromosomes 6S and 12 L, respectively (Myint et al. 2012). Like *Bph14*, *Bph26* encodes a CC-NB-LRR protein that mediates antibiosis to BPH. Sequence comparison indicated that *Bph26* is the same as *Bph2*, which was overcome by biotype 2. However, pyramiding of *Bph25* and *Bph26* could significantly improve BPH resistance, suggesting a valuable application in rice resistance breeding (Tamura et al. 2014).

*Bph17* was cloned from Sri Lankan *indica* variety Rathu Heenati. Initially, *Bph17* was fine-mapped to a 79 kb region containing four clustered genes on chromosome 4S. Transgenic tests showed that three genes independently confer resistance to BPH, and gene pyramided transgenic lines showed enhanced resistance. *Bph17* is actually a cluster of three genes encoding plasma membrane-localized lectin receptor kinases (*OsLecRK1*—*OsLecRK3*), which collectively function to confer broad-spectrum, durable resistance and provide an important gene source for MAS and transgenic breeding for BPH resistance (Liu et al. 2015).

*bph29*, a recessive gene from *O. rufipogon*, fine-map to a 24 Kb region on chromosome 6S. Through a transgenic experiment, the *bph29* allele from the susceptible variety was transferred into the resistant variety, and the positive progenies were susceptible, whereas the negative progenies retained high resistance. *bph29* encodes a B3 DNA-binding protein. Expression patterns analysis showed that *bph29* is restricted to the vascular tissue where BPH attacks. Expression of *bph29* activates the SA signaling pathway and suppresses the jasmonic acid/ethylene (JA/Et)-dependent pathway after BPH infestation and induces callose deposition in phloem cells, resulting in antibiosis to BPH (Wang et al. 2015).

#### Genes and TFs associated with BPH resistance

In addition to the traditional map-based cloning method, some genes and transcription factors (TFs) associated with BPH resistance have been identified through reverse genetics approaches such as T-DNA mutants and genes homology. *Bphi008a* is a resistance gene that is induced by BPH feeding; it is involved in ethylene signaling. Plants carrying a transgenic *Bphi008a* allele show significantly enhanced resistance to BPH (Hu et al. 2011b). Another two genes, *OsERF3* and *OsHI-LOX*, are ethylene response factors and lipoxygenase genes, respectively, involved in a JA/Et-dependent pathway and act as inhibitor of the gene expression to improve resistance to BPH (Lu et al. 2011; Zhou et al. 2009). With the development of rice genomics and proteomics, continued screening and validation of genes that are regulated by BPH feeding, and clarification of resistance mechanisms will promote research of BPH—associated genes and offer possibilities for resistance breeding.

### Molecular breeding for BPH resistance

Since the 1970s, several BPH resistance genes such as *Bph1*, *bph2*, *Bph3* and *bph4* have been identified and transferred into elite susceptible varieties at IRRI, and a series of improved cultivars (e.g. IR26, IR36, IR50 and IR72) with BPH resistance were developed and released (Jairin et al. 2007a; Jena and Kim, 2010). However, the improved cultivars carrying single resistance gene lose effectiveness due to the evolution of new biotypes (Jena and Kim 2010). Therefore, to develop new varieties with more durable and stable BPH resistance, there has to be use of more genes, preferably pyramided into multiple gene lines or possibly deployed in multiline single gene mixtures such that new biotypes will be hampered or delayed.

#### Integrating MAS into conventional rice breeding

**Fig. 4:**
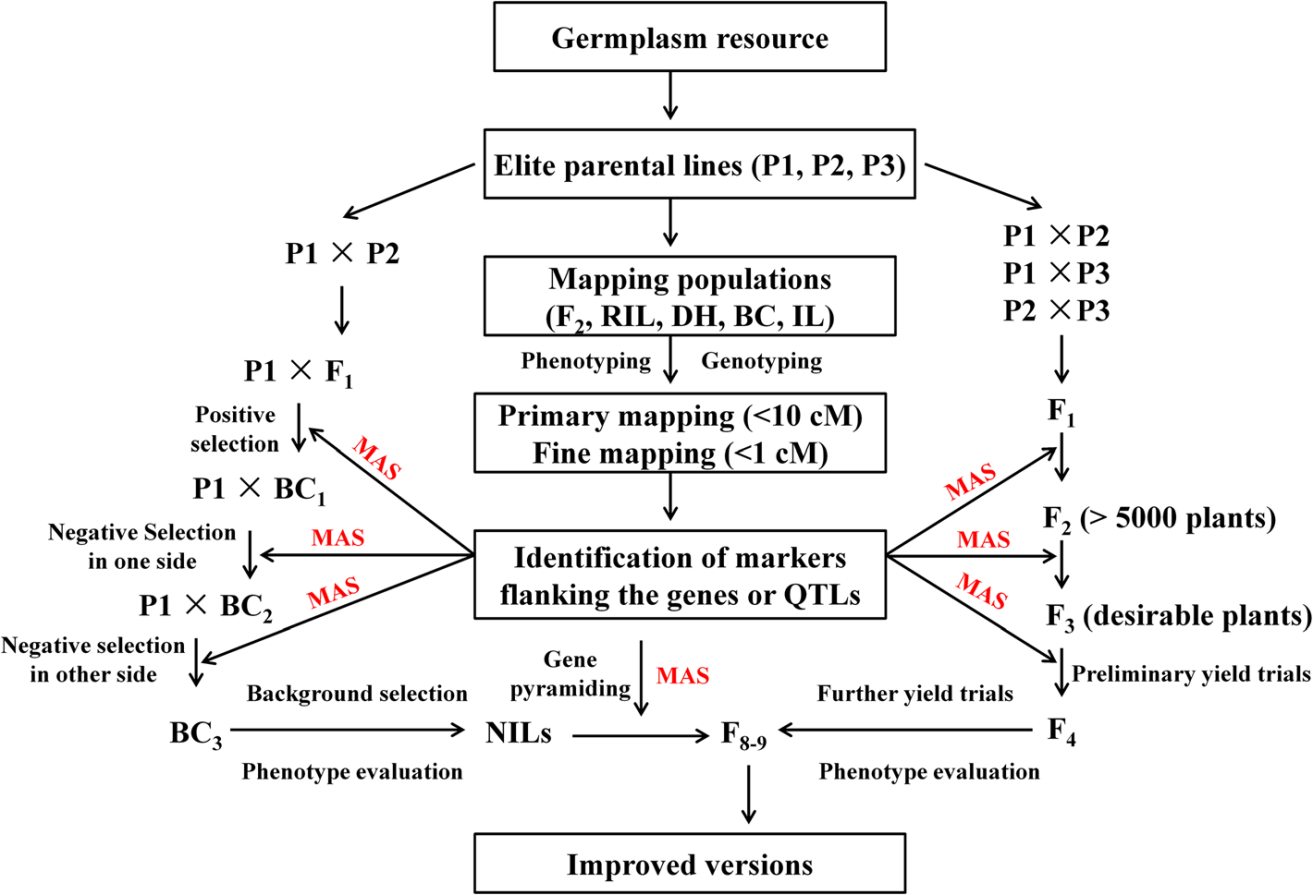
An integrated strategy of MAS and conventional breeding. MAS strategy is in the center position throughout the entire process of breeding. The primary goal is development of useful markers tightly linked to target QTLs/genes by QTL mapping experiments (primary mapping, fine mapping and QTL validation). MABC include three generations of backcrosses and one generation of selfing, accompanied by positive and negative selection for minimizing the donor segments linked to target gene, and background selection for maximizing the recurrent genome. After phenotype evaluation of BC_3_F_2_ lines, NILs containing single target gene are obtained. Multiple NILs that carrying different genes are crossed each other to produce pyramided lines. MAS based conventional breeding include 8–9 generations of selfing, accompanied by multiple cross within three parents, field and MAS selection in a large F_2_ population, preliminary and further yield trials in F_3_ and F_4-8_ population. After phenotype evaluation, the F_8-9_ progenies with enhanced target traits and high yield potential could be obtained, designated as ‘improved versions’

MAS greatly increases the efficiency and effectiveness of breeding. By determining and developing DNA markers for target genomic regions, desired individuals possessing particular genes or QTLs can be identified in germplasm collections based on genotyping rather than phenotyping (Collard et al. 2005). New strategy that fits breeder requirements should include a planned MAS strategy, MAS-based backcrossing breeding (MABC) and gene pyramiding (Fig. 4).

#### Construction of a MAS system for using BPH resistance genes

The efficiency of MAS largely depends on the distance between molecular markers and genes/QTLs associated with target traits. The development of useful markers tightly linked to target traits is accomplished by QTL mapping experiments. Generally, the markers are validated in fine mapping studies. Based on the positional information of BPH-resistance genes previously reported (Table 2), SSR and InDel markers adjacent to related genes were designed, and used to track the target genes in the segregating generation, and to test whether these markers were closely linked with genes. Thus, several MAS systems with high efficiency associated with these genes were developed (Table 3).

**Table 3:** Markers used in MAS for BPH resistance in rice

Marker	chr	position	QTL/gene	F(5′–3′)	R(5′–3′)	Reference
c3-14	3	35646876	*QBph3*	GGCAAAATTAGACGGCACG	GAATATGCATTTTGTTTGGAG	Hu et al. 2015a
IN76-2	3	35689799	*Bph14*	CTGCTGCTGCTCTCGTATTG	CAGGGAAGCTCCAAGAACAG	Du et al. 2009
RM261	4	6579056	*Bph15,QBph4.1*	CTACTTCTCCCCTTGTGTCG	TGTACCATCGCCAAATCTCC	Hu et al. 2015a
g12140-2	4	6691854	*Bph15*	ACCAAACACGGTGGATGAGA	AATGGAAAAGAGGAGGACAACAG	Lv et al. 2014
xc4-27	4	6899420	*Bph15,QBph4.1*	GCATAAGCGCCCTAGCC	GCTAGTTGCAGGCACGC	Hu et al. 2015a
20 M14	4	6900345	*Bph15*	ATGCTGACGGTGCTAGGAGT	CAGTCCATCCACACAACTTGA	Lv et al. 2014
RH7	4	6949655	*Bph17*	CTTGCGTTCCGTAGGAGAAG	TGAGTGTAACCCGAAGTGGC	Liu et al. 2015
RHC10	4	6972108	*Bph17*	CAATACGGGAGATTTGGAGT	TTGGGAAGCATACGAGTGA	Liu et al. 2015
IN156	4	7006594	*Bph15, Bph17*	AGGTGAAGCTGATGTGCTTG	CGATACTTATTGCAACACAC	Hu et al. 2012
B43	4	8760137	*Bph20*	ACTCCAATTGGTTCCTGTGG	TGGACTAAAAGCCGATGAGC	Rahman et al. 2009
RM119	4	21414516	*Bph6*	CATCCCCCTGCTGCTGCTGCTG	CGCCGGATGTGTGGGACTAGCG	Qiu et al. 2010
S00310	6	214474	*Bph25*	CAACAAGATGGACGGCAAGG	TTGGAAGAAAAGGCAGGCAC	Myint et al. 2012
RM589	6	1381865	*Bph3*	ATCATGGTCGGTGGCTTAAC	CAGGTTCCAACCAGACACTG	Jairin et al. 2009
RM260	12	19549286	*Bph10*	ACTCCACTATGACCCAGAG	GAACAATCCCTTCTACGATCG	Ishii et al. 1994
RM313	12	20872949	*Bph10*	TGCTACAAGTGTTCTTCAGGAC	GCTCACCTTTTGTGTTCCAC	Ishii et al. 1994
RM463	12	22125823	*Bph2*	TTCCCCTCCTTTTATGGTGC	TGTTCTCCTCAGTCACTGCG	Sun et al. 2006
RM6869	12	22253179	*Bph2*	GAGCTCCTTGTAGTGACCCG	ATCAGCCTCGCCAGCTTC	Sun et al. 2006
RM6217	12	22671954	*Bph9*	CGCAGATGGAGATTCTTGAAGG	ACAGCAGCAAGAGCAAGAAATCC	Su et al. 2006
IN187	12	22875241	*Bph18,Bph9*	GACCCCCTTCGAGTCTAAGAAC	CTTCTTTGAACTCATAGACAG	Hu et al. 2013
7312.T4	12	22885300	*Bph18*	ACGGCGGTGAGCATTGG	TACAGCGAAAAGCATAAAGAGTC	Jena et al. 2006
RM3331	12	23494476	*Bph18*	CCTCCTCCATGAGCTAATGC	AGGAGGAGCGGATTTCTCTC	Suh et al. 2011
RM5479	12	24356237	*Bph21,Bph26*	AACTCCTGATGCCTCCTAAG	TCCATAGAAACAATTTGTGC	Myint et al. 2012
B121	12	24202618	*Bph21*	CGTCGTACATTCTGAAATGGAG	GGACATGGAGATGGTGGAGA	Rahman et al. 2009

#### MABC for BPH resistance

**Fig. 5:**
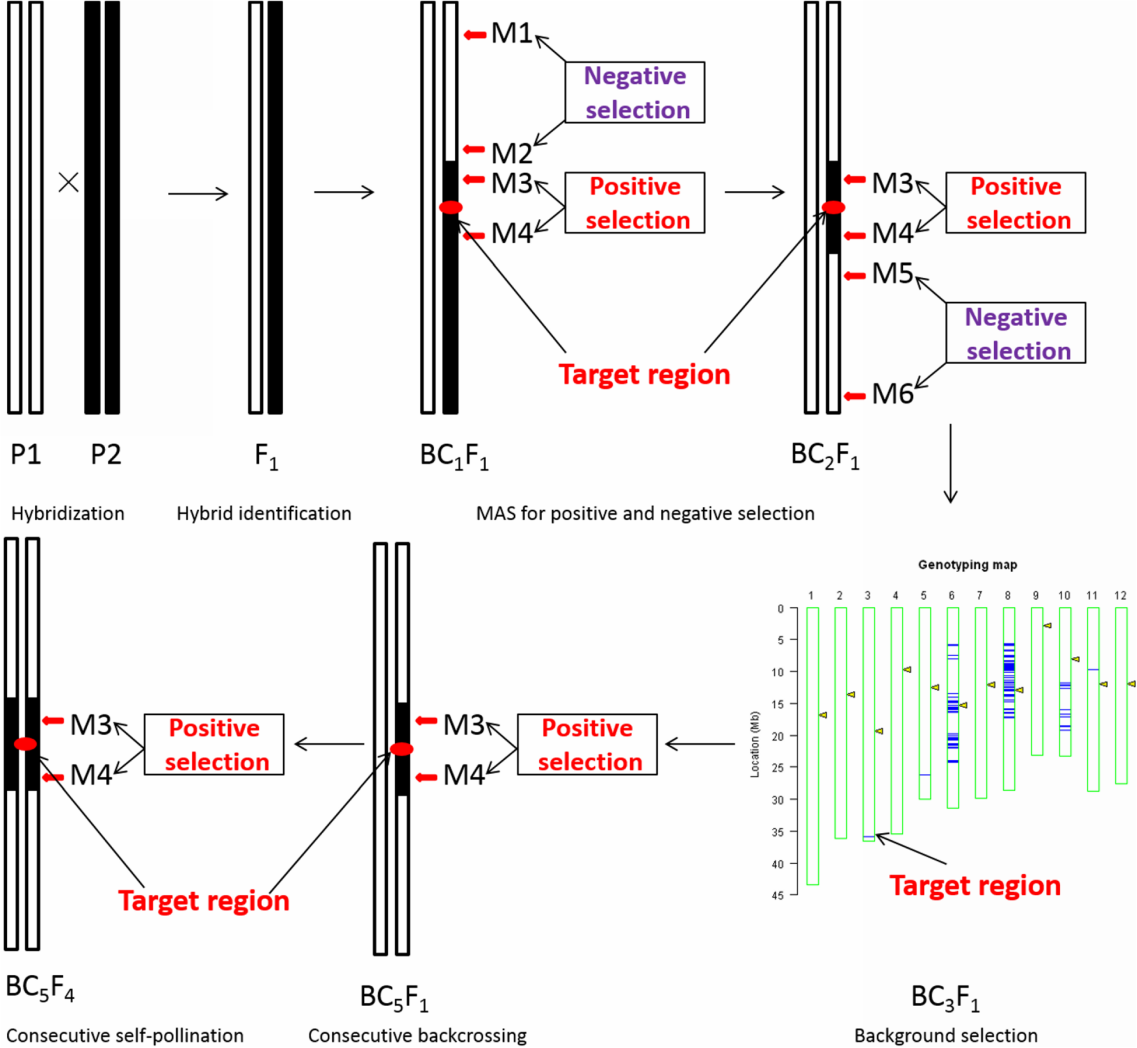
A flowchart for marker assisted backcross breeding (MABC). *M3* and *M4* are markers for positive selection of target genes. *M1*, *M2*, *M5* and *M6* are linked markers for negative selection of linked segments of target genes

It takes a minimum of 6–8 backcrosses to fully recover a recurrent parent genome using conventional breeding methods, but MABC enables the procedure to be shortened to 3 or 4 backcrosses (Tanksley et al. 1989). There are three levels of selection in which markers are applied in backcross breeding (Fig. 5). Firstly, markers are used to select target alleles whose effects are difficult to observe phenotype (e.g. resistance in the absence of actual disease/pest tests), this is referred to as ‘positive selection’; secondly, markers are used to select for progeny with the target gene and tightly-linked flanking markers in order to produce chromosomes that harbor the target allele with minimal surrounding DNA from the donor parent (minimizing linkage drag), designated as ‘negative selection’ or ‘recombinant selection’; thirdly, markers that are distributed across all 12 rice chromosomes can be selected for recovery of the recurrent parent genome, known as ‘background selection’. A typical example of MABC that used markers for all three objectives was performed by Chen et al. (2000).

MABC has been used to develop multiple BPH-resistance introgressions (ILs) or near-isogenic lines (NILs). Using a *Bph18*-cosegergation marker 7312. T4A for positive selection, and 260 SSR markers across all rice 12 chromosomes for background selection, *Bph18* was transferred into an elite *japonica* variety ‘Junambyeo’ and ILs with enhanced BPH resistance were developed (Suh et al. 2011). Using negative selection, linkage drag between *Bph3* and *Wx*^*a*^ alleles was successfully broken resulting in ILs with broad spectrum BPH resistance and good quality (Jairin et al. 2009). In our laboratory, a number of genes (*Bph3*, *Bph6*, *Bph9*, *Bph14*, *Bph15*, *Bph10*, *Bph18*, *Bph20*, *QBph4*, *QBph3*) were individually incorporated into 9311 (an elite variety in China) using MABC, and a set of NILs was developed with enhanced BPH resistance. These NILs harbor target gene regions of less than 100 kb and the recurrent parent genome (>99.5 %) was recovered with a breeding chip with high-density SNP markers for negative and background selection (data unpublished).

#### Pyramiding BPH resistance genes

Using MAS, we can simply and easily combine multiple genes/QTLs together into a single genotype simultaneously. Through conventional breeding to pyramid traits, individual plants/lines must be phenotypically screened for all tested traits. However, it may be impossible or extremely difficult to pyramid traits such as pest resistance where unique biotypes may be needed for screening as the presence of one gene may prevent phenotypic selection for others. Pyramiding of resistance genes or QTLs in rice has now become an effective method for developing lines with disease and pest resistance (Divya et al. 2014; Dokku et al. 2013; Jiang et al. 2015; Pradhan et al. 2015; Singh et al. 2015; Suh et al. 2013; Wan et al. 2014).

Using MAS based conventional breeding, progress has been made in pyramiding two or more major BPH resistance genes into susceptible cultivars. The pyramided lines (PLs) carrying *Bph1* and *bph2* genes showed higher resistance than the lines with only *bph2* (Sharma et al. 2004). Qiu et al. (2012) used MAS for pyramiding *Bph6* and *Bph12* genes into *japonica* and *indica* cultivars. The PLs had stronger resistance level than ILs with *Bph6* alone, followed by the single-*Bph12* ILs. In addition, three dominant BPH resistance genes *(Bph14*, *Bph15*, *Bph18*) were pyramided into the elite *indica* rice 9311 and its hybrids using MABC. The results showed an additive effect of those pyramiding genes, the order of the gene effect being 14/15/18 ≥ 14/15 > 15/18 ≥ 15 > 14/18 ≥ 14 ≥ 18 > none (Hu et al. 2013). Additionally, pyramiding BPH resistance genes and other resistances have become routine in rice breeding. Wan et al. (2014) reported development of a new elite restorer line possessing tolerance to BPH, stem borer, leaf folder and herbicide through pyramiding *Bph14*, *Bph15*, *Cry1C*, and glufosinate-resistance gene *bar*.

In order to develop new cultivars with durable BPH resistance, we should not only use gene pyramiding, but exploit genetic diversity for ecological reasons. Zhu et al. (2000) reported an example of genetic diversity and blast disease control in rice. Furthermore, multiple NILs were developed representing all possible combinations of several blast resistance QTLs/genes from a durably resistant cultivar (Fukuoka et al. 2015; Khanna et al. 2015). Similarly, we have pyramided *Bph14* and *Bph15* into several different rice hybrids, and experiments indicated that planting resistant pyramided hybrids around conventional susceptible hybrids significantly reduced the overall population of BPH over a large field area, thereby reducing the BPH threat and contributing to sustainable of rice production (Hu et al. 2011a). Moreover, multilines (NILs, ILs, or PLs) carrying different assortments of genes should also help in containing BPH populations to manageable levels.

### Conclusion and perspective

In the recent years, significant progress has been made in molecular breeding of rice for yield, quality, biotic and abiotic stress resistances and certain agronomic traits (Rao et al. 2014). However, the genetics rice: BPH interaction and molecular breeding for BPH resistance have been restrained due to the intricacy of interaction between rice and BPH. Host—plant resistance is an effective environmentally friendly approach to control BPH and maintain yield potential of cultivars (Jena and Kim, 2010). Future breeding approaches must focus on developing cultivars with durable, broad-spectrum resistance.

The first objective is to identify and characterize new resistance genes from diverse germplasm resources, particularly wild species. The second objective is to understand the molecular interactions between rice and BPH. We should not only accelerate research on map-base cloning of BPH resistance genes, but also pay attention to and the genome and genetics of BPH itself. The BPH genome was sequenced and genomes of BPH and its endosymbionts revealed complex complementary contributions for host adaptation (Xue et al. 2014). Mapping of the rice resistance-breaking gene of the BPH has facilitated understanding of interactions of BPH and rice (Jing et al. 2014; Kobayashi et al. 2014). The third objective is to pyramid major genes or QTLs or to deploy NILs or ILs carrying multiple single resistance genes in multilines. Recently, molecular breeding design (MDB) have become popular for molecular breeding in crop improvement and should contribute to future breeding outcomes (Xu and Zhu, 2012). Molecular breeding designs for BPH resistance will involve three steps: (1) map all QTLs for BPH resistance by high-throughput genotyping and reproducible phenotyping; (2) evaluate and reconfirm allelic variation in these QTLs by development of NILs; and (3) conduct design breeding according to a bioinformatics platform and simulation studies. The final objective is to develop new varieties that contain the best genotypic combinations to confer durable resistance.
